# Identifying Parameters to Distinguish Non-Diabetic Renal Diseases from Diabetic Nephropathy in Patients with Type 2 Diabetes Mellitus: A Meta-Analysis

**DOI:** 10.1371/journal.pone.0064184

**Published:** 2013-05-14

**Authors:** Shuang Liang, Xue-Guang Zhang, Guang-Yan Cai, Han-Yu Zhu, Jian-Hui Zhou, Jie Wu, Pu Chen, Shu-peng Lin, Qiang Qiu, Xiang-Mei Chen

**Affiliations:** Department of Nephrology, State Key Laboratory of Kidney Diseases, Chinese PLA General Hospital, Beijing, China; University of Washington, United States of America

## Abstract

**Background:**

Renal injuries in patients with diabetes include diabetic nephropathy (DN) and non-diabetic renal diseases (NDRD). The value of a clinical diagnosis of DN and NDRD remains inconclusive. We conducted a meta-analysis of the literature to identify predictive factors of NDRD and to compare the clinical characteristics of DN and NDRD for differential diagnosis.

**Methods:**

We searched PubMed (1990 to January 2012), Embase (1990 to February 2009), and CNKI (1990 to January 2012) to identify studies that enrolled patients with DN and NDRD. Then, the quality of the studies was assessed, and data were extracted. The results were summarized as odds ratios (ORs) for dichotomous outcomes and weighted mean differences (WMDs) for continuous outcomes.

**Results:**

Twenty-six relevant studies with 2,322 patients were included. The meta-analysis showed that the absence of diabetic retinopathy (DR) predicts NDRD (OR, 0.15; 95% confidence interval [CI], 0.09–0.26, *p*<0.00001). A shorter duration of diabetes mellitus (DM) also predicted NDRD (weighted mean difference, −34.67; 95% CI, −45.23–−24.11, *p*<0.00001). The levels of glycosylated hemoglobin (HbA1C%), blood pressure (BP), and total cholesterol were lower in patients with NDRD, whereas triglycerides and body mass index were higher. Other clinical parameters, including age, 24-h urinary protein excretion, serum creatinine, creatinine clearance, blood urea nitrogen, and glomerular filtration rate were not different between patients with NDRD and DN.

**Conclusions:**

We identified that the absence of DR, shorter duration of DM, lower HbA1C, and lower BP may help to distinguish NDRD from DN in patients with diabetes. This could assist clinicians in making a safe and sound diagnosis and lead to more effective treatments.

## Introduction

Diabetic nephropathy (DN) is one of the major complications of diabetes mellitus (DM). It is estimated that 20–40% of patients with DM will develop a diabetic renal disease. DN is the leading cause of chronic kidney disease and end-stage renal disease worldwide [1], [2]. The diagnosis of DN is almost always based on clinical grounds and supported by persistent proteinuria, hypertension, and a progressive decline in renal function. The validity of this clinical approach is well established in patients with type 1 diabetes but not in those with type 2 diabetes [3]. Furthermore, non-diabetic renal diseases (NDRD) such as minimal change disease or idiopathic membranous nephropathy, either isolated or superimposed on an underlying DN, have been reported. The prevalence of biopsy-proven NDRD in patients with diabetes varies from 10–85% among reports [4]–[7]. These differences may be due to selection criteria, biopsy threshold, or the populations being studied [8], [9].

Treatments for DN and NDRD are quite different. Many NDRD lesions can be treated with immunosuppressants other than the standard angiotensin-converting enzyme inhibitors (ACEIs) or angiotensin receptor blockers (ARBs). Thus, it is important to distinguish NDRD from DN early. A kidney biopsy is necessary to confirm the diagnosis, but is invasive. Nephrologists are sometimes reluctant to perform a renal biopsy on patients with DM because of the potential risks of the procedure such as hematuria, perirenal hematoma, arterial embolization, and even the necessity for a nephrectomy [10]. Moreover, there exist some contraindications for renal biopsy such as the solitary kidney and cortical atrophy [11]. Additionally, many primary hospitals are at present unable to perform the renal biopsy. Therefore, nephrologists must provide a suspected diagnosis using the clinical and laboratory data available before a biopsy is performed. Zhou *et al.* [12] constructed a diagnostic model with good sensitivity (90%) and specificity (92%) based on a logistic regression analysis. Only diabetes duration, systolic blood pressure (SBP), glycosylated hemoglobin (HbA1c), hematuria, and diabetic retinopathy (DR) showed statistical significance. Other studies have reported factors to distinguish NDRD from DN only after renal histology is available. However, the results were not uniform, which was likely due to differences in the study populations or selection criteria; thus, a systematic assessment of published findings is needed. Therefore, we conducted a meta-analysis of case-control studies to investigate the potential roles of clinical and laboratory data for discriminating NDRD and DN in patients with type 2 diabetes.

## Materials and Methods

### Searching

The searching was performed by two reviewers independently (S.L. and X.G.Z.). Electronic databases, including PubMed (1990 to January 2012), Embase (1990 to February 2009), and CNKI (1990 to January 2012) were searched following the modified version of the Meta-analysis Of Observational Studies in Epidemiology (MOOSE) guidelines [13]. We also scanned the references of eligible studies, the “Web of Knowledge Cited References” list, and the “Related Articles” link in PubMed to identify potentially relevant studies. Hand searching of textbooks and unpublished materials such as conference proceedings was also used. The following search terms were employed: diabetic nephropathy, non-diabetic nephropathy, diabetes mellitus AND kidney disease, diabetic kidney disease, and biopsy. No race restriction was utilized.

### Study Selection and Quality Assessment

All studies describing diabetic kidney diseases were of interest. Titles and/or abstracts of all citations were screened by S.L. and X.G.Z., and relevant original studies were read in full. This search resulted in 63 studies in the quality assessment. Studies were included if they met the following criteria: 1. Retrospective case-control, cross-sectional and prospective studies, but not other types, such as case reports. 2. Studies that provided detailed clinical and laboratory parameters at renal biopsy. Exclusion criteria were: 1. The diagnosis of DN or NDRD was based on a clinical diagnosis rather than biopsy. 2. Studies that included type 1 diabetes or did not specify the type of diabetes. 3. Studies that did not adopt appropriate statistical methods. 4. Studies with obvious publication bias (lack of negative studies) were excluded by simply not being published. We assessed other potential biases in the published literature as a reason for exclusion. For example, we excluded patients with other kidney diseases such as drug-induced renal disease; with acute complications such as ketoacidosis and urinary infection; with serious cardiovascular, liver, pancreatic disease, and psychopathy; with malignant tumors and secondary DM.

### Data Extraction

Data were extracted by one reviewer (S.L.) and independently verified by another (G.Y.C). Information extracted included lead author, publication year, country of data collection, sample characteristics (*e*.*g*., age, sex, and sample size), and study design type. Clinical and laboratory parameters included presence or absence of hematuria and retinopathy, age, diabetes duration, blood pressure, blood urea nitrogen (BUN), serum creatinine (Scr), 24-h proteinuria estimation, and others at the time of renal biopsy.

Most of the studies set minimum criteria for what constituted biopsy evidence of diabetes or NDRD. DN was diagnosed based on the presence of mesangial expansion and diffuse intercapillary glomerulosclerosis with or without Kimmelstiel–Wilson nodules, basement membrane thickening, and/or exudative lesions, such as a fibrin cap, capsular drop, or hyaline thrombus [14].

### Statistical methods

All analyses were undertaken with RevMan 5.1. Heterogeneity across the included studies was analyzed using the heterogeneity χ^2^ (Cochrane Q) statistic and the I^2^ test. I^2^ values >25, 50, and 75% were considered evidence of mild, moderate, and severe statistical heterogeneity, respectively. We applied both fixed and random-effects models. If significant heterogeneity was present (*p*<0.05), a random-effects model was used. Otherwise (*p*>0.05), a fixed-effects model was used. Statistical significance was set at *p*<0.05 [15]. Categorical variables were analyzed using the odds ratios (ORs) and associated 95% confidence intervals (CIs). OR explains the probability of presence/absence of a condition in association with NDRD. Continuous variables were expressed as means ± standard deviations and analyzed using the weighted mean differences (WMDs) and 95% CIs [15]. In several included studies, patients were grouped as group I, isolated DN; group II, isolated NDRD; and group III, NDRD with underlying diabetic glomerulosclerosis. Perhaps given the treatment, some studies regarded DN+NDRD as NDRD, and did not provide the exact number and original clinical data of the isolated NDRD. In this meta-analysis, we also regarded groups II and III as NDRD. Thus, all patients were grouped into one of two categories: DN or NDRD. We adjusted for the pooled variance using the formula S^2^  =  (n_1_−1) S_1_^2^ + (n_2_−1) S_2_^2^/(n_1_ + n_2_−2) [16].

## Results

**Figure 1:**
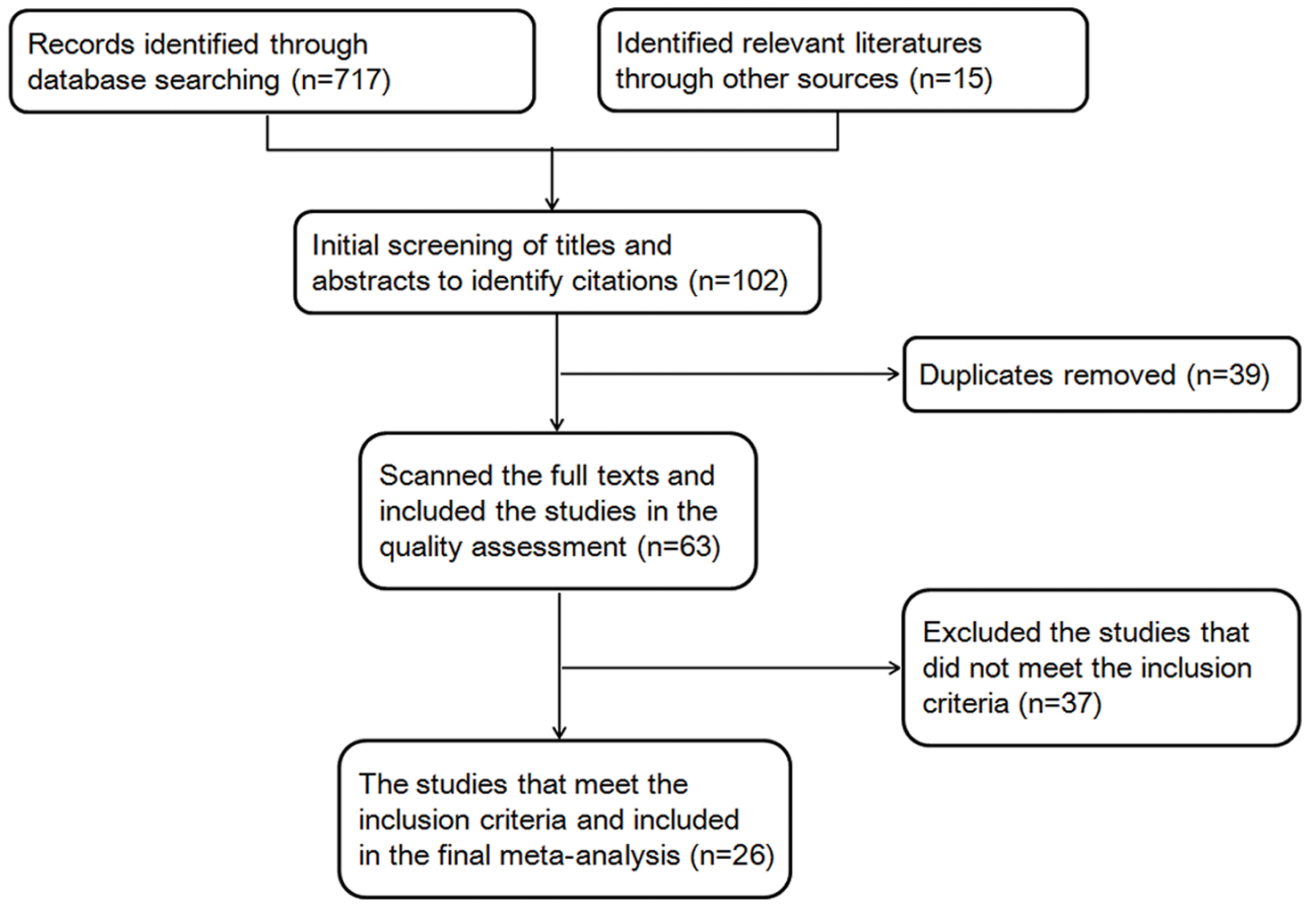
Flow chart showing study selection process for the meta-analysis.

We initially identified 732 potentially relevant studies. Based on the scanning of the titles and abstracts, 630 articles were excluded. After reading the full text of the remaining studies and references, 26 studies [3], [5], [6], [8], [11], [12], [17]–[36] were included in the final analysis (Fig. 1), involving 2,322 patients: 1,156 with DN and 1,166 with NDRD. Data extracted from the 26 studies are presented in Table S1. The studies included were from different regions: two from the USA, two from Denmark, two from Italy, one from Austria, and the others from Asia. Twenty-two studies were retrospective, and the other four were prospective case-control studies. Sample size ranged from 22 to 333. We performed subgroup and sensitivity analyses when necessary to evaluate the effect of heterogeneity among studies. Comparisons of clinical and laboratory features at renal biopsy of the 26 studies are summarized in Table S2. ORs, WMDs, and 95% CIs of all studies are reported.

### DN Risk Factors

#### **Hematuria**

In two studies (studies 1 and 11), the presence of hematuria was defined as ≥2 red blood cells per high-power field in a centrifuged urine sample prior to biopsy, whereas in three studies (studies 8, 9, and 10) it was defined as >3 red blood cells per high power field. In other studies, microscopic hematuria was defined as >10 red blood cells/µL (studies 2, 4, 15, and 17) and >5 red blood cells/µL (study 26) on phase-contrast urine microscopy. Other studies did not specify the definition of hematuria. A random-effects model was used based on the test for heterogeneity: χ^2^ = 56.49, df = 15 (*p*<0.0001), I^2^ = 73%. The estimated pooled ORs showed that the presence of hematuria was in closer association with NDRD than DN (OR, 2.05; 95% CI, 1.25–3.36, *p* = 0.004). Given that the majority of the studies included were conducted in Asian populations, a sub-group analysis of the Asian population was performed, and results were similar.

#### **DR**

**Figure 2:**
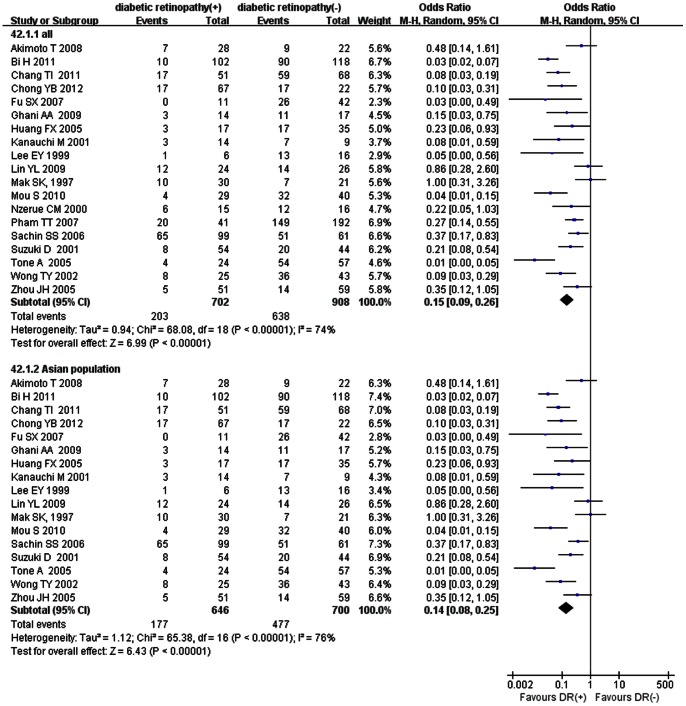
Meta-analysis of non-diabetic renal diseases (NDRD) and diabetic neuropathy (DN) associated with diabetic retinopathy (DR).

DR was diagnosed on ophthalmoscopy and fluorescence retinography by an ophthalmologist, based on the presence of background retinopathy (microaneurysms, hemorrhages, and soft or hard exudates) with or without proliferative changes. The meta-analysis suggested that patients with DR were less likely to have NDRD than to have DN. (OR, 0.15; 95% CI, 0.09–0.26, *p*<0.00001). A sensitivity analysis, including only those conducted in Asian populations, was carried out (OR, 0.14; 95% CI, 0.08–0.25, *p*<0.00001) (Fig. 2). Therefore, the presence of DR seemed to be a useful index for identification of DN, while the absence of DR seemed to be predictive of NDRD.

### Comparison of Clinical and Laboratory Parameters in Patients with DN and NDRD

#### **DM Duration**

**Figure 3:**
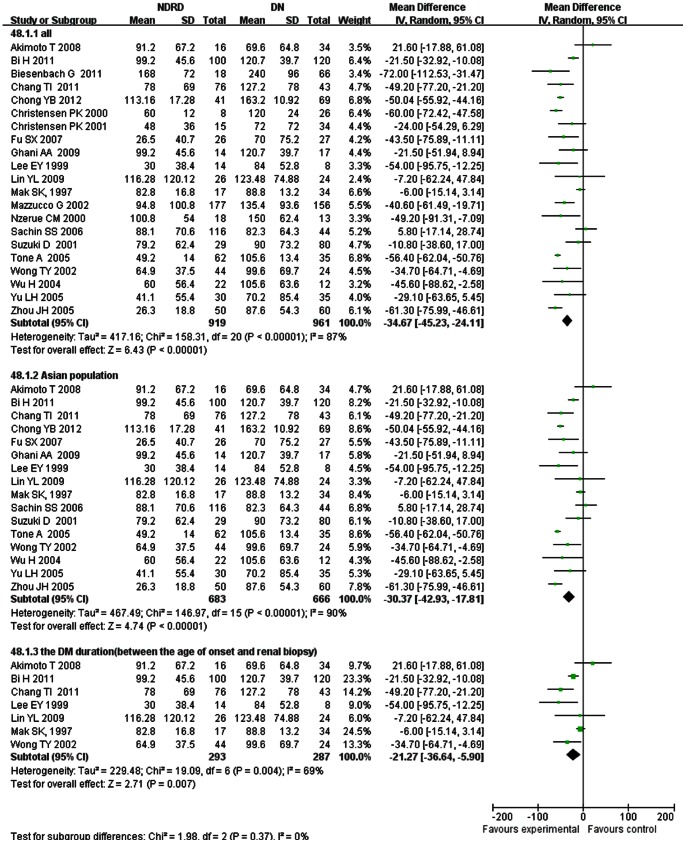
Comparison of DM duration between patients with non-diabetic renal disease (NDRD) and diabetic neuropathy (DN).

DM duration of patients with NDRD was significantly shorter than that of patients with DN (WMD, −34.67; 95% CI, −45.23–−24.11, *p*<0.00001). The result for the Asian population was similar (WMD, −30.37; 95% CI, −42.93–−17.81, *p*<0.00001). However, the definition of DM duration differed among studies. In seven studies, DM duration was the period between the onset of diabetes and renal biopsy. In another two studies, it was regarded as the duration between the onset of diabetes and when renal diseases were detected. The definition of DM duration was not described in other studies. A sensitivity analysis, including the aforementioned seven studies, was carried out. The result was similar (OR, −21.27; 95% CI, −36.64–−5.90, *p* = 0.007), with moderate heterogeneity (χ^2^ = 19.09, df = 6, I^2^ = 69%) (Fig. 3).

#### **HbA1C% Level**

In both overall and the Asian population, the HBA1c of patients with NDRD was slightly lower than that of patients with DN (WMD, −0.39; 95% CI, −0.58–−0.21, *p*<0.0001 and WMD, −0.50; 95% CI, −0.69–−0.30, *p*<0.00001, respectively).

#### **Blood Pressure**

In most of the studies (studies 1, 4, 8, 9, 15, 18, and 19), hypertension was defined as SBP>140 mmHg and/or diastolic blood pressure (DBP)>90 mmHg or taking antihypertensive drugs, and in study 10 it was defined as SBP≥130 mmHg and DBP≥80 mmHg. In two other studies, arterial hypertension was diagnosed according to the World Health Organization criteria: SBP≥160 mmHg and/or DBP≥95 mmHg, or if antihypertensive medication was being prescribed.

**Figure 4:**
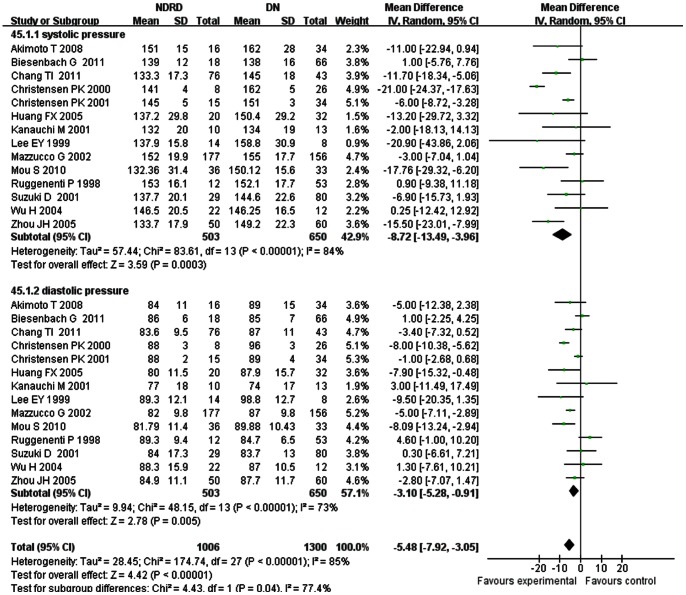
Comparison of blood pressure between patients with non-diabetic renal disease (NDRD) and diabetic neuropathy (DN).

Subgroup analyses were performed according to SBP and DBP. SBP and DBP of patients with NDRD were both lower (SBPs: WMD, −8.72; 95% CI, −13.49–−3.96; *p* = 0.0003; DBPs: WMD, −3.10; 95% CI, −5.28–−0.91, *p* = 0.005) than those in patients with NDRD (Fig. 4).

#### **24-h Urine Total Protein Excretion**

A random-effects model was used due to obvious heterogeneity among the studies (heterogeneity: χ^2^ = 414.59, df = 16, I^2^ = 96%). No significant difference in the 24-h urine total protein excretion (g/24 h) was detected between patients with NDRD and DN (WMD, −0.61; 95% CI, −1.53–0.31, *p* = 0.20). The sub-group analysis of the Asian population showed similar results (WMD, −0.81; 95% CI, −1.90–0.28, *p* = 0.14).

#### **Renal Function**

We assessed renal function using four sub-studies, including the level of Scr, creatinine clearance (Ccr), glomerular filtration rate (GFR), and BUN. The Scr, Ccr, GFR, and BUN were not significantly different between patients with DNRD and DN (WMD, 2.99; 95% CI, −22.12–28.10, *p* = 0.82; WMD, 3.21; 95% CI, −12.10–18.53, *p* = 0.68; WMD, 2.30; 95% CI, −4.08–8.68, *p* = 0.48; WMD, −0.93; 95% CI, −4.21–2.35, *p* = 0.58, respectively).

#### **Lipid Profile and Body Mass Index (BMI)**

The findings suggested that patients with NDRD tended to have slightly higher triglycerides (mmol/L) than those with DN (WMD, 0.85; 95% CI, 0.26–1.44, *p* = 0.005), as well as a higher BMI (kg/m^2^) (WMD, 1.54; 95% CI, 0.91– 2.17, *p*<0.00001). However, patients with NDRD tended to have a slightly lower total cholesterol (mmol/L) than that of patients with DN (WMD, −0.40; 95% CI, −0.65–−0.15, *p* = 0.002). Further studies with larger sample size are needed to clarify these differences.

## Discussion

The analyses showed that patients with hematuria were more likely to develop NDRD. Erythrocyte casts and dysmorphic erythrocytes in the urine sediment may be more specific than microhematuria for discriminating NDRD from DN. However, only four of the twenty-six included studies mentioned the occurrence of erythrocyte casts and dysmorphic erythrocytes. Thus, more studies are needed to further evaluate the role of casts and dysmorphic erythrocytes.

Our study confirmed the accepted view that DR is an important predictor of DN. However, DR mostly precedes the progression of DN in patients with type 1 diabetes, but this sequence is often inconsistent in patients with type 2 diabetes [37]–[41]. Although the prevalence of DR was significantly higher in patients with DN compared to patients with NDRD, 23.6% of patients with biopsy-proven DN did not have retinopathy. In contrast, no evidence of DN was present in 17.6% of patients with DR [40], [42], [43]. Taken together, the lack of DR is predictive of NDRD, but not an exclusion criterion for DN.

No significant difference was found between the two groups of age at the time of biopsy. Diabetes duration of patients with NDRD was shorter than that of patients with DN. But type 2 diabetes may have developed long before these patients were diagnosed. Therefore, the known diabetes duration does not accurately predict the presence or severity of DN [17]. HbA1C was lower in patients with NDRD than those with DN. Hyperglycemia may promote kidney damage through a hemodynamic effect, glycosylation, the polyol pathway, or oxidative stress [44].

One accepted view is that hypertension is an independent risk factor for DN. The mechanisms of hypertension in DM remain complex, such as stimulation of the sympathetic nervous system or activation of the renin-angiotensin system resulting in water-sodium retention. Our analyses showed that SBPs and DBPs were both lower in patients with NDRD. In the studies included, study 19 and 25 found that among DN patients, antihypertensive treatment could reduce the rate of decline in GFR and thereby postponed ESRD. But study 21 found no difference in the rate of decline in GFR between patients treated with or without ACEI or ARBs both in DN and NDRD patients. However, anti-hypertensive treatment was initiated and increased at different time points, and a variety of anti-hypertensive drugs were used during follow-up, all of which may induce a bias in evaluating the effects of these medications. And only one of the included studies mentioned the lipid-lowering treatment. So it is hard to draw a conclusion that blood pressure and lipid profiles were different between NDRD and DN patients.

In terms of renal function, our meta-analysis demonstrated that the Scr, Ccr, GFR, and BUN at renal biopsy were not significantly different between patients with DN and NDRD. The impact of NDRD on renal outcomes in type2 diabetic patients has not been well established, and most of the available data were based on the results of previous cross- sectional studies [45], [46]. In a study [47] that compared the rate of renal decline in diabetic and non-diabetic patients with chronic kidney disease (GFR<50 ml/min), when controlling for albuminuria, the mean slope of renal decline was similar in patients with and without diabetes. Higher albuminuria was a predictor of poorer renal outcome, regardless of diabetes condition. Among the studies included in this meta-analysis, five were prospective observational studies, and only two of them provided the number of patients entering the end point. As was shown in the Table S2, patients with NDRD had a lower risk to develop ESRD than patients with DN, but the P value did not reach statistical significance (p = 0.07). What's more, the definition of end point in these two studies was different. One regarded ESRD as an advanced renal failure requiring maintenance dialysis, or Scr≥700 µmol/l, while the other one defined ESRD as Scr >500 µmol/L, which may result in a bias. The renal outcome in patients with DM varied, and depended on the specific type of non-diabetic renal lesion. The limited information or the small number of each subtype in the included studies did not permit further subgroup analyses. Moreover, most of patients with NDRD were treated with immunosuppressive agents, which likely had a positive effect on the renal outcomes. Thus, more prospective studies focusing on the renal outcome in patients with DN and NDRD are needed.

In summary, the main findings of this meta-analysis were clinical predictors that facilitate to discrimination of NDRD from DN: (1) absence of DR. (2) shorter DM duration, (3) lower HbA1C, and (4) lower blood pressure. We found no difference in the ages, the 24-h urine total protein excretion, Scr, Ccr, GFR, or BUN of patients with NDRD and DN.

The limitations of this study should be discussed, since meta-analyses have inherent limitations. Because the studies were mainly observational in nature, the statistical combination of the data might have been subject to selection and reporting biases [48]. But we had eliminated bias from our analysis where possible by establishing a strict methodology and a predefined quality assessment process. However, heterogeneity remained between studies in terms of potential confounders such as age and sex. Furthermore, studies of histological patterns were associated with the bias of indication and the experience of physicians in conducting renal biopsies. We agree with the viewpoint that development of a logistic regression diagnostic model would be helpful to distinguish NDRD from DN. However, Meta-analysis is conducted basically on study-level summary data, and original clinical and laboratory parameters of each patient are not available. So we are not able to build a regression model, which maybe another limitation of meta-analysis.

This study advocates for a higher suspicion of a NDRD in patients with type 2 diabetes. We also identified important features that discriminate between NDRD and DN. This could assist clinicians in making a safe and sound diagnosis and lead to more effective medical management.

## Supporting Information

Table S1 **Characteristics of the 26 Studies Included in the Meta-analysis.** **Abbreviations**: DM, diabetes mellitus; DR, Diabetic Retinopathy; BP, blood pressure; SBP, systolic blood pressure; DBP, diastolic blood pressure; HbA1C, hemoglobin A1C; Scr, serum creatinine; Ccr, creatinine clearance; TG, triglyceride; TC, total cholesterol; GFR, glomerular filtration rate; BUN, blood urine nitrogen; UA, serum uric acid; BMI, body mass index; Hb, hemoglobin; ALB, serum albumin; TP, serum total protein; FBG, fasting blood-glucose; PBG, postprandial blood glucose; NS, nephrotic syndrome; 24h-u, 24-hour urine protein excretion; Hb, hemoglobin; R, retrospective case-control study; P: prospective case-control study. **Note:** a, The study was performed in Caucasian, African American, Asian and Hispanic population. b, The study was performed in Malay, Chinese and Indian population. c, The study was performed in African Americans.
(DOC)

Table S2 **Comparisons of clinical and laboratory features at renal biopsy of the 26 studies.** **Note:** a, categorical variable; b, continuous variable. I^2^ statistic as a measure of heterogeneity. * Sensitivity analysis included seven studies in which DM duration was the period between the onset of diabetes and renal biopsy. # Renal outcome was assessed by the risk to develop ESRD. **Abbreviations:** OR, odd ratio; CI, confidence interval; WMD, weighted mean difference; DR, Diabetic Retinopathy; DM, diabetes mellitus; HbA1C, hemoglobin A1C; SBP, systolic blood pressure; DBP, diastolic blood pressure; Scr, serum creatinine; Ccr, creatinine clearance; GFR, glomerular filtration rate; BUN, blood urine nitrogen; TG, triglyceride; TC, total cholesterol; BMI, body mass index; Hb, hemoglobin. **Conversion factors for units:** Scr in mg/dL to µmol/L, ×88.4; BUN in mg/dL to mmol/L, ×0.357; TG in mg/dL to mmol/L, ×0.01129; TC in mg/dL to mmol/L, ×0.02586.
(DOC)

## References

[1] Kikkawa R, Koya D, Haneda M (2003) Progression of diabetic nephropathy. Am J Kidney Dis 41: S19–21. 12612945 10.1053/ajkd.2003.50077

[2] Ritz E, Rychlik I, Locatelli F, Halimi S (1999) End-stage renal failure in type 2 diabetes: A medical catastrophe of worldwide dimensions. Am J Kidney Dis 34: 795–808. 10561134 10.1016/S0272-6386(99)70035-1

[3] Sachin SS, Gowrishankar S, Kishan AG, Anuradha R (2006) Nondiabetic renal disease in type 2 diabetic mellitus. Nephrology 11: 533–537. 17199793 10.1111/j.1440-1797.2006.00681.x

[4] Olsen S, Mogensen CE (1999) How often is NIDDM complicated with non-diabetic renal disease? An analysis of renal biopsies and the literature. Diabetologia 39: 1638–1645. 10.1007/s001250050628 8960856

[5] Lee EY, Chung CH, Choi SO (1999) Non-diabetic renal disease in patients with non-insulin dependent diabetes mellitus. Yonsei Med J 40: 321–326. 10487133 10.3349/ymj.1999.40.4.321

[6] Nzerue CM, Hewan-Lowe K, Harvey P, Mohammed D, Furlong B, et al (2000) Prevalence of non-diabetic renal disease among African-American patients with type II diabetes mellitus. Scand J Urol Nephrol 34: 331–335. 11186474 10.1080/003655900750048378

[7] Prakash J, Sen D, Usha, Kumar NS (2001) Non-diabetic renal disease in patients with type 2 diabetes mellitus. J Assoc Physicians India 49: 415–420. 11762610

[8] Huang F, Yang Q, Chen L, Tang S, Liu W, et al (2007) Renal pathological change in patients with type 2 diabetes is not always diabetic nephropathy: a report of 52 cases. Clin Nephrol 67: 293–297. 17542338 10.5414/cnp67293

[9] Bertani T, Mecca G, Sacchi G, Remuzzi G (1986) Superimposed nephritis: a separate entity among glomerular disease? Am J Kidney Dis 7: 205–212. 2937291 10.1016/s0272-6386(86)80004-x

[10] Whittier WL, Korbet SM (2004) Renal biopsy: update. Curr Opin Nephrol Hypertens 13: 661–665. 15483458 10.1097/00041552-200411000-00013

[11] Ghani AA, Al Waheeb S, Al Sahow A, Hussain N (2009) Renal biopsy in patients with type 2 diabetes mellitus: indications and nature of the lesions. Ann Saudi Med 29: 450–453. 19847082 10.4103/0256-4947.57167 PMC2881432

[12] Zhou J, Chen X, Xie Y, Li J, Yamanaka N, et al (2008) A differential diagnostic model of diabetic nephropathy and non-diabetic renal diseases. Nephrol Dial Transplant 23: 1940–1945. 18156459 10.1093/ndt/gfm897

[13] Stroup DF, Berlin JA, Morton SC, Olkin I, Williamson GD, et al (2000) Meta analysis of observational studies in epidemiology: A proposal for reporting. Meta-analysis Of Observational Studies in Epidemiology (MOOSE) Group. JAMA 283: 2008–2012. 10789670 10.1001/jama.283.15.2008

[14] Fioretto P, Mauer M (2007) Histopathology of diabetic nephropathy. Semin Nephrol 27: 195–207. 17418688 10.1016/j.semnephrol.2007.01.012 PMC2746982

[15] Zhong WS, Wu YL, Gu LJ (2003) Review Manager (RevMan) – a bridge leading the clinicians to meta analysis. The Journal of Evidence – Based Medicine 3: 234–246.

[16] Gönen M (2010) The Bayesian *t*-test and beyond. Methods Mol Biol 620: 179–199. 20652504 10.1007/978-1-60761-580-4_4

[17] Tone A, Shikata K, Matsuda M, Usui H, Okada S, et al (2005) Clinical features of non-diabetic diseases in patients with type 2 diabetes. Diabetes Res Clin Pract 69: 237–242. 16098920 10.1016/j.diabres.2005.02.009

[18] Suzuki D, Takano H, Toyoda M, Umezono T, Uehara G, et al (2001) Evaluation of renal biopsy samples of patients with diabetic nephropathy. Intern Med 40: 1077–1084. 11757760 10.2169/internalmedicine.40.1077

[19] Fu SX, Pei HY, Xing LL, Li SM, Yang L (2007) The study of renal biopsy indicator for 2 diabetic mellitus patients with renal damage. J Clin Intern Med 24: 38–40.

[20] Biesenbach G, Bodlaj G, Pieringer H, Sedlak M (2011) Clinical versus histological diagnosis of diabetic nephropathy–is renal biopsy required in type 2 diabetic patients with renal disease? QJM 104: 771–774. 21504987 10.1093/qjmed/hcr059

[21] Bi H, Chen N, Ling G, Yuan S, Huang G, et al (2011) Nondiabetic renal disease in type 2 diabetic patients: a review of our experience in 220 cases. Ren Fail 33: 26–30. 21219202 10.3109/0886022X.2010.536292

[22] Chang TI, Park JT, Kim JK, Kim SJ, Oh HJ, et al (2011) Renal outcomes in patients with type 2 diabetes with or without coexisting non-diabetic renal disease. Diabetes Res Clin Pract 92: 198–204. 21320734 10.1016/j.diabres.2011.01.017

[23] Pham TT, Sim JJ, Kujubu DA, Liu IL, Kumar VA (2007) Prevalence of nondiabetic renal disease in diabetic patients. Am J Nephrol 27: 322–328. 17495429 10.1159/000102598

[24] Wu H, Xu ZW, Chen H, Fang F, Lin FR, et al (2004) Differentiation on concomitant of non-diabetic renal disease from diabetic nephropathy in type 2 diabetes Mellitus. J Clin Intern Med 21: 125–127.

[25] Yu LH, Ren ST, Guo R, Xu CF, Li HL, et al (2005) Study on pathological and epidemiological characteristics of non-diabetic renal diseases in patients with type2 diabetes mellitus. Chin J Endocrinol Metab 21: 61–62.

[26] Mak SK, Gwi E, Chan KW, Wong PN, Lo KY, et al (1997) Clinical predictors of non-diabetic renal disease in patients with non-insulin dependent diabetes mellitus. Nephrol Dial Transplant 12: 2588–2591. 9430856 10.1093/ndt/12.12.2588

[27] Chong YB, Keng TC, Tan LP, Ng KP, Kong WY, et al (2012) Clinical predictors of non-diabetic renal disease and role of renal biopsy in diabetic patients with renal involvement: a single centre review. Ren Fail 34: 323–328. 22250665 10.3109/0886022X.2011.647302

[28] Akimoto T, Ito C, Saito O, Takahashi H, Takeda S, et al (2008) Microscopic hematuria and diabetic glomerulosclerosis – clinicopathological analysis of type 2 diabetic patients associated with overt proteinuria. Nephron Clin Pract 109: c119–126. 18663323 10.1159/000145454

[29] Kanauchi M, Dohi K (2001) Predictors of diabetic renal lesions in type 2 diabetes associated with microalbuminuria. Eur J Clin Invest 31: 110–112. 11168447 10.1046/j.1365-2362.2001.00761.x

[30] Wong TY, Choi PC, Szeto CC, To KF, Tang NL, et al (2002) Renal outcome in type 2 diabetic patients with or without coexisting nondiabetic nephropathies. Diabetes Care 25: 900–905. 11978688 10.2337/diacare.25.5.900

[31] Christensen PK, Larsen S, Horn T, Olsen S, Parving HH (2001) Renal function and structure in albuminuric type 2 diabetic patients without retinopathy. Nephrol Dial Transplant 16: 2337–2247. 11733625 10.1093/ndt/16.12.2337

[32] Ruggenenti P, Gambara V, Perna A, Bertani T, Remuzzi G (1998) The nephropathy of non-insulin-dependent diabetes: predictors of outcome relative to diverse patterns of renal injury. J Am Soc Nephrol 9: 2336–2343. 9848788 10.1681/ASN.V9122336

[33] Mazzucco G, Bertani T, Fortunato M, Bernardi M, Leutner M, et al (2002) Different patterns of renal damage in type 2 diabetes mellitus: a multicentric study on 393 biopsies. Am J Kidney Dis 39: 713–720. 11920336 10.1053/ajkd.2002.31988

[34] Mou S, Wang Q, Liu J, Che X, Zhang M, et al (2010) Prevalence of non-diabetic renal disease in patients with type 2 diabetes. Diabetes Res Clin Pract 87: 354–359. 20005594 10.1016/j.diabres.2009.11.012

[35] Christensen PK, Gall MA, Parving HH (2000) Course of glomerular filtration rate in albuminuric type 2 diabetic patients with or without diabetic glomerulopathy. Diabetes Care 23: B14–20. 10860186

[36] Lin YL, Peng SJ, Ferng SH, Tzen CY, Yang CS (2009) Clinical indicators which necessitate renal biopsy in type 2 diabetes mellitus patients with renal disease. Int J Clin Pract 63: 1167–1176. 18422591 10.1111/j.1742-1241.2008.01753.x

[37] Andersen AR, Christiansen JS, Andersen JK, Kreiner S, Deckert T (1983) Diabetic nephropathy in Type 1 (insulin-dependent) diabetes: an epidemiological study. Diabetologia 25: 496–501. 6363177 10.1007/BF00284458

[38] Mogensen CE, Christensen CK (1984) Predicting diabetic nephropathy in insulin-dependent patients. N Engl J Med 311: 89–93. 6738599 10.1056/NEJM198407123110204

[39] Mauer SM, Chavers BM, Steffes MW (1990) Should there be an expanded role for kidney biopsy in the management of patients with type I diabetes? Am J Kidney Dis 16: 96–100. 2382660 10.1016/s0272-6386(12)80561-0

[40] Parving HH, Gall MA, Skøtt P, Jørgensen HE, Løkkegaard H, et al (1992) Prevalence and causes of albuminuria in non-insulin-dependent diabetic patients. Kidney Int 41: 758–762. 1513098 10.1038/ki.1992.118

[41] Gall MA, Rossing P, Skøtt P, Damsbo P, Vaag A, et al (1991) Prevalence of micro- and macroalbuminuria, arterial hypertension, retinopathy and large vessel disease in European type 2 (non-insulin-dependent) diabetic patients. Diabetologia 34: 655–661. 1955098 10.1007/BF00400995

[42] Kveder R, Kajtna-Koselj M, Rott T, Bren AF (2001) Nephrotic syndrome in patients with diabetes mellitus is not always associated with diabetic nephropathy. Nephrol Dial Transplant 16: 86–87. 11568254 10.1093/ndt/16.suppl_6.86

[43] Christensen PK, Larsen S, Horn T, Olsen S, Parving HH (2000) Causes of albuminuria in patients with type 2 diabetes without diabetic retinopathy. Kidney Int 58: 1719–1731. 11012906 10.1046/j.1523-1755.2000.00333.x

[44] United Kingdom Prospective Diabetes Study Group (1995) United Kingdom Prospective Diabetes Study (UKPDS).13: Relative efficacy of randomly allocated diet, sulphonylurea, insulin, or metformin in patients with newly diagnosed non-insulin dependent diabetes followed for three years. Br Med J 310: 83–88. 7833731 PMC2548496

[45] Bertani T, Mecca G, Sacchi G, Remuzzi G (1986) Superimposed nephritis: a separate entity among glomerular diseases? Am J Kidney Dis 7: 205–212. 2937291 10.1016/s0272-6386(86)80004-x

[46] Orfila C, Lepert JC, Modesto A, Pipy B, Suc JM (1998) IgA nephropathy complicating diabetic glomerulosclerosis. Nephron 79: 279–287. 9678427 10.1159/000045050

[47] Lorenzo V, Saracho R, Zamora J, Rufino M, Torres A (2012) Similar renal decline in diabetic and non-diabetic patients with comparable levels of albuminuria. Nephrol Dial Transplant 25: 835–841. 10.1093/ndt/gfp475 19762600

[48] Egger M, Schneider M, Davey Smith G (1998) Spurious precision? Meta-analysis of observational studies. BMJ 316: 140–144. 9462324 10.1136/bmj.316.7125.140 PMC2665367

