# Supplementary material for: Identifying Parameters to Distinguish Non-Diabetic Renal Diseases from Diabetic Nephropathy in Patients with Type 2 Diabetes Mellitus: A Meta-Analysis
Source: PLoS One. 2013 May 14;8(5):e64184. doi: 10.1371/journal.pone.0064184 (PMC3653878; doi:10.1371/journal.pone.0064184)
Supplement: Table S1 — Characteristics of the 26 Studies Included in the Meta-analysis. Abbreviations: DM, diabetes mellitus; DR, Diabetic Retinopathy; BP, blood pressure; SBP, systolic blood pressure; DBP, diastolic blood pressure; HbA1C, hemoglobin A1C; Scr, serum creatinine; Ccr, creatinine clearance; TG, triglyceride; TC, total cholesterol; GFR, glomerular filtration rate; BUN, blood urine nitrogen; UA, serum uric acid; BMI, body mass index; Hb, hemoglobin; ALB, serum albumin; TP, serum total protein; FBG, fasting blood-glucose; PBG, postprandial blood glucose; NS, nephrotic syndrome; 24h-u, 24-hour urine protein excretion; Hb, hemoglobin; R, retrospective case-control study; P: prospective case-control study. Note: a, The study was performed in Caucasian, African American, Asian and Hispanic population. b, The study was performed in Malay, Chinese and Indian population. c, The study was performed in African Americans. (DOC) [file pone.0064184.s001.doc]

**Table 1. Characteristics of the 26 Studies Included in the Meta-analysis**

| **Study** | **Lead author** | **Year of publication** | **Country** | **Type of study** | **No. of DN** | **No. of NDRD** | **The information abstracted from the article** |
| --- | --- | --- | --- | --- | --- | --- | --- |
| **1** | [Ghani AA](http://www.ncbi.nlm.nih.gov/pubmed?term=%22Ghani%20AA%22%5BAuthor%5D) | 2009 | Kuwait | R | 17 | 14 | Age, sex, DM duration, Scr, GFR, 24h-u; the number of patients with [hematuria](http://www.iciba.com/hematuria/), DR, hypertension |
| **2** | [Tone A](http://www.ncbi.nlm.nih.gov/pubmed?term=%22Tone%20A%22%5BAuthor%5D) | 2005 | Japan | R | 35 | 62 | Age, sex, HbA1C, TP, Scr, 24h-u; the number of patients with hematuria, DR |
| **3** | [Suzuki D](http://www.ncbi.nlm.nih.gov/pubmed?term=%22Suzuki%20D%22%5BAuthor%5D) | 2001 | Japan | R | 80 | 29 | Age, sex, DM duration, SBP, DBP, HbA1C, Scr,24h-u; the number of patients with [hematuria](http://www.iciba.com/hematuria/), DR |
| **4** | [Lee EY](http://www.ncbi.nlm.nih.gov/pubmed?term=%22Lee%20EY%22%5BAuthor%5D) | 1999 | Korea | R | 8 | 14 | Age, sex, DM duration, BP, HbA1C, Scr, Ccr, BUN, TC, TG, BMI, 24h-u; the number of patients with DR, [hematuria](http://www.iciba.com/hematuria/), proteinuria, hypertension |
| **5** | Fu SX | 2007 | China | R | 27 | 26 | Age, DM duration, BMI; the number of patients with DR |
| **6** | [Biesenbach G](http://www.ncbi.nlm.nih.gov/pubmed?term=%22Biesenbach%20G%22%5BAuthor%5D) | 2011 | Austria | R | 66 | 18 | Age, sex, DM duration, SBP, DBP, HbA1C, BMI; the number of patients with hypertension |
| **7** | Huang FX | 2007 | China | R | 32 | 20 | Age, sex, DM duration, SBP, DBP, HbA1C, Scr, FBG, TC, TG, BUN, 24h-u; the number of patients with hematuria, DR, hypertension, proteinuria |
| **8** | [Bi H](http://www.ncbi.nlm.nih.gov/pubmed?term=%22Bi%20H%22%5BAuthor%5D) | 2011 | China | R | 120 | 100 | Age, DM duration, Scr, GFR, 24h-u; the number of patients with DR, [hematuria](http://www.iciba.com/hematuria/), proteinuria, hypertension, hyperlipidemia, hyperuricemia |
| **9** | Sachin SS | 2006 | India | R | 44 | 116 | Age, sex, DM duration, Scr, the number of patients with DR, [hematuria](http://www.iciba.com/hematuria/), proteinuria, hypertension |
| **10** | [Chang TI](http://www.ncbi.nlm.nih.gov/pubmed?term=%22Chang%20TI%22%5BAuthor%5D) | 2011 | Korea | R | 43 | 76 | Age, sex, DM duration, BP, HbA1C, Scr, Ccr, Hb, TC,24h-u; the number of patients with [hematuria](http://www.iciba.com/hematuria/), DR, proteinuria, hypertension |
| **11** | [Pham TT](http://www.ncbi.nlm.nih.gov/pubmed?term=%22Pham%20TT%22%5BAuthor%5D) | 2007 | USA^a^ | R | 64 | 169 | Age, sex, race, Scr, GFR, 24h-u; the number of patients with hematuria, DR, neuropathy |
| **12** | Wu H | 2004 | China | R | 12 | 22 | DM duration, SBP, DBP, Scr, Ccr; the number of patients with [hematuria](http://www.iciba.com/hematuria/), DR, hypertension |
| **13** | Yu LH | 2005 | China | R | 35 | 30 | Age, sex, DM duration; the number of patients with hypertension, NS, renal failure |
| **14** | Zhou JH | 2005 | China | R | 60 | 50 | Age, DM duration, SBP, DBP, HbA1C, Scr, BUN, UA, TG, TC, ALB, 24h-u; the number of patients with [hematuria](http://www.iciba.com/hematuria/),DR, hypertension, hyperlipidemia, hyperuricemia, cardiopathy, NS |
| **15** | [Mak SK](http://www.ncbi.nlm.nih.gov/pubmed?term=%22Mak%20SK%22%5BAuthor%5D) | 1997 | Hong Kong | R | 34 | 17 | Age, sex, DM duration, HbA1C, Scr, Ccr, BUN, UA, 24h-u; the number of patients with [hematuria](http://www.iciba.com/hematuria/), DR, proteinuria, hypertension, cardiopathy |
| **16** | Chong YB | 2012 | Malaysia ^b^ | R | 69 | 41 | Age, sex, race, DM duration, Scr, ALB, HbA1C, 24h-u; the number of patients with [hematuria](http://www.iciba.com/hematuria/),DR |
| **17** | Akimoto T | 2008 | Japan | R | 34 | 16 | Age, sex, DM duration, Scr, Ccr, ALB, HbA1C, SBP, 24h-u; the number of patients with [hematuria](http://www.iciba.com/hematuria/),DR |
| **18** | Kanauchi M | 2001 | Japan | R | 13 | 10 | Age, sex, DM duration, Scr, GFR, HbA1C, SBP, DBP, 24h-u; ALB; the number of patients with DR, hypertension |
| **19** | Wong TY | 2002 | Hong Kong | P | 24 | 44 | Age, sex, DM duration, Scr, BMI, TC, 24h-u; ALB; the number of patients with hematuria, hypertension, DR, proteinuria |
| **20** | Nzerue CM | 2000 | USA ^c^ | R | 13 | 18 | Age, sex, DM duration, Scr, HbA1C; ALB, 24h-u |
| **21** | Christensen PK | 2001 | Denmark | P | 34 | 15 | Age, sex, DM duration,HbA1C,TC,BMI; the number of patients with hypertension; |
| **22** | Ruggenenti P | 1998 | Italy | P | 53 | 12 | Age, sex, DM duration,HbA1C,SBP, DBP,HbA1C,Scr |
| **23** | Mazzucco G | 2001 | Italy | R | 156 | 177 | Age, sex, DM duration, SBP, DBP, Scr,24h-u |
| **24** | Mou S | 2010 | China | R | 33 | 36 | Age, SBP, DBP, Scr, GFR, FBG, HbA1C,24h-u; the number of patients with DR |
| **25** | Christensen PK | 2000 | Denmark | P | 26 | 8 | Age, sex, SBP, DBP, Scr, GFR, BMI, HbA1C, TC,24h-u; the number of patients with hypertension |
| **26** | [Lin YL](http://www.ncbi.nlm.nih.gov/pubmed?term=Lin%20YL%5BAuthor%5D&cauthor=true&cauthor_uid=18422591) | 2009 | Taiwan | R | 24 | 26 | Age, sex,Scr,Ccr,BUN,HbA1C, DM duration, 24h-u; the number of patients with hematuria, DR, proteinuria |

**Abbreviations**: DM, diabetes mellitus; DR, Diabetic Retinopathy; BP, blood pressure; SBP, systolic blood pressure; DBP, diastolic blood pressure; HbA1C, hemoglobin A1C; Scr, serum creatinine; Ccr, creatinine clearance; TG, triglyceride; TC, total cholesterol; GFR, glomerular filtration rate; BUN, blood urine nitrogen; UA, serum uric acid; BMI, body mass index; Hb, hemoglobin; ALB, serum albumin; TP, serum total protein; FBG, fasting blood-glucose; PBG, postprandial blood glucose; NS, nephrotic syndrome; 24h-u, 24-hour urine protein excretion; Hb, hemoglobin; R, retrospective case-control study; P: prospective case-control study.

**Note:** a, The study was performed in Caucasian, African American, Asian and Hispanic population.

b, The study was performed in Malay, Chinese and Indian population.

c, The study was performed in African Americans.
