# Supplementary material for: Identifying Parameters to Distinguish Non-Diabetic Renal Diseases from Diabetic Nephropathy in Patients with Type 2 Diabetes Mellitus: A Meta-Analysis
Source: PLoS One. 2013 May 14;8(5):e64184. doi: 10.1371/journal.pone.0064184 (PMC3653878; doi:10.1371/journal.pone.0064184)
Supplement: Table S2 — Comparisons of clinical and laboratory features at renal biopsy of the 26 studies. Note: a, categorical variable; b, continuous variable. I2 statistic as a measure of heterogeneity. * Sensitivity analysis included seven studies in which DM duration was the period between the onset of diabetes and renal biopsy. # Renal outcome was assessed by the risk to develop ESRD. Abbreviations: OR, odd ratio; CI, confidence interval; WMD, weighted mean difference; DR, Diabetic Retinopathy; DM, diabetes mellitus; HbA1C, hemoglobin A1C; SBP, systolic blood pressure; DBP, diastolic blood pressure; Scr, serum creatinine; Ccr, creatinine clearance; GFR, glomerular filtration rate; BUN, blood urine nitrogen; TG, triglyceride; TC, total cholesterol; BMI, body mass index; Hb, hemoglobin. Conversion factors for units: Scr in mg/dL to µmol/L, ×88.4; BUN in mg/dL to mmol/L, ×0.357; TG in mg/dL to mmol/L, ×0.01129; TC in mg/dL to mmol/L, ×0.02586. (DOC) [file pone.0064184.s002.doc]

**Table S2. Comparisons of clinical and laboratory features at renal biopsy of the 26 studies.**

| **Clinical & biochemical parameters** | **No. of studies** | **No. of DN** | **No. of NDRD** | **χ^2^** | **I^2^** | **ORs or WMDs** | **95% CI** | ***P* Value** |
| --- | --- | --- | --- | --- | --- | --- | --- | --- |
| **Hematuria^a^** | 16 | 700 | 816 | 56.49 | 73% | OR, 2.05 | 1.25–3.36 | 0.004 |
| **Hematuria in the Asian population** | 15 | 636 | 647 | 53.17 | 74% | OR, 2.13 | 1.25–3.62; | 0.005 |
| **DR^a^** | 19 | 774 | 884 | 68.08 | 74% | OR, 0.15 | 0.09–0.26; | < 0.00001 |
| **DR in the Asian population** | 17 | 697 | 697 | 65.38 | 76% | OR, 0.14 | 0.08–0.25 | < 0.00001 |
| **Age^b^** | 24 | 1084 | 1094 | 420.03 | 95% | WMD,- 0.72 | -3.22– 1.78 | 0.57 |
| **Age of the Asian population** | 17 | 672 | 677 | 404.75 | 96% | WMD, -1.28 | -4.59–2.03 | 0.45 |
| **DM duration (month) ^b^** | 21 | 961 | 919 | 158.31 | 87% | WMD, -34.67 | -45.23–-24.11 | < 0.00001 |
| **DM duration in the Asian population** | 16 | 666 | 683 | 146.97 | 90% | WMD, -30.37 | -42.93–-17.81 | < 0.00001 |
| **DM duration in seven studies*** | 7 | 287 | 293 | 19.09 | 69% | WMD, -21.27 | -36.64–-5.90 | 0.007 |
| **HbA1C (%)^b^** | 17 | 657 | 468 | 33.27 | 52% | WMD, -0.39 | -0.58– -0.21 | < 0.00001 |
| **HbA1C of the Asian population** | 12 | 465 | 397 | 17.87 | 38% | WMD, -0.50 | -0.69–-0.30 | < 0.00001 |
| **SBP(mmHg)^b^** | 14 | 650 | 503 | 83.61 | 84% | WMD, -8.72 | -13.49– -3.96 | 0.0003 |
| **DBP(mmHg)^b^** | 14 | 650 | 503 | 48.15 | 73% | WMD, -3.10 | -5.28–-0.91 | 0.005 |
| **Proteinuria (g/24 h) ^b^** | 17 | 875 | 877 | 414.59 | 96% | WMD, -0.61 | -1.53–0.31 | 0.20 |
| **Proteinuria in the Asian population** | 13 | 589 | 501 | 391.10 | 97% | WMD, -0.81 | -1.90–0.28 | 0.14 |
| **Scr (µmol/L)^b^** | 19 | 931 | 1025 | 235.74 | 92% | WMD, 2.99 | -22.12–28.10 | 0.82 |
| **Ccr (mL/min)^b^** | 5 | 130 | 154 | 17.90 | 78% | WMD, 3.21 | -12.10–18.53 | 0.68 |
| **GFR (mL/min/1.73 m^2^)^b^** | 6 | 281 | 344 | 21.05 | 76% | WMD, 2.30 | -4.08–8.68 | 0.48 |
| **BUN (mmol/L)^b^** | 5 | 158 | 127 | 36.84 | 89% | WMD, -0.93 | -4.21–2.35 | 0.58 |
| **TG (mmol/L)^b^** | 3 | 100 | 84 | 4.17 | 52% | WMD, 0.85 | 0.26–1.44 | 0.005 |
| **TC (mmol/L)^b^** | 7 | 227 | 227 | 5.97 | 50% | WMD, -0.40 | -0.65–-0.15 | 0.002 |
| **BMI (kg/m^2^)^b^** | 6 | 185 | 125 | 7.36 | 32% | WMD, 1.54 | 0.91–2.17; | < 0.00001 |
| **Hb^b^** | 2 | 103 | 126 | 0.07 | 0% | WMD,22.69 | 17.10–28.28 | < 0.00001 |
| **Renal outcome^#^** | 2 | 50 | 49 | 0.61 | 0% | WMD,0.49 | 0.23– 1.06 | 0.07 |

**Note:** a, categorical variable; b, continuous variable.

I^2^ statistic as a measure of heterogeneity.

* Sensitivity analysis included seven studies in which DM duration was the period between the onset of diabetes and renal biopsy.

# Renal outcome was assessed by the risk to develop ESRD.

**Abbreviations:** OR, odd ratio; CI, confidence interval; WMD, weighted mean difference; DR, Diabetic Retinopathy; DM, diabetes mellitus; HbA1C, hemoglobin A1C; SBP, systolic blood pressure; DBP, diastolic blood pressure; Scr, serum creatinine; Ccr, creatinine clearance; GFR, glomerular filtration rate; BUN, blood urine nitrogen; TG, triglyceride; TC, total cholesterol; BMI, body mass index; Hb, hemoglobin.

**Conversion factors for units:**

Scr in mg/dL to µmol/L, ×88.4; BUN in mg/dL to mmol/L, ×0.357;

TG in mg/dL to mmol/L, ×0.01129; TC in mg/dL to mmol/L, ×0.02586
